# A model for boundary-driven tissue morphogenesis

**DOI:** 10.1073/pnas.2505160122

**Published:** 2025-09-18

**Authors:** Daniel S. Alber, Shiheng Zhao, Alexandre O. Jacinto, Eric F. Wieschaus, Stanislav Y. Shvartsman, Pierre A. Haas

**Affiliations:** ^a^Department of Chemical and Biological Engineering, Princeton University, Princeton, NJ 08540; ^b^The Lewis-Sigler Institute for Integrative Genomics, Princeton University, Princeton, NJ 08540; ^c^Max Planck Institute for the Physics of Complex Systems, Dresden 01187, Germany; ^d^Max Planck Institute of Molecular Cell Biology and Genetics, Dresden 01307, Germany; ^e^Center for Systems Biology Dresden, Dresden 01307, Germany; ^f^Center for Computational Biology, Flatiron Institute, Simons Foundation, New York, NY 10010; ^g^Department of Molecular Biology, Princeton University, Princeton, NJ 08540

**Keywords:** morphogenesis, *Drosophila* development, mechanical bifurcation, tissue mechanics

## Abstract

Tissue deformations during morphogenesis can be active, driven by internal processes, or passive, resulting from stresses applied at their boundaries. Here, we introduce the *Drosophila* hindgut primordium as a model for studying boundary-driven tissue morphogenesis. We characterize its deformations and show that its complex shape changes can be a passive consequence of the deformations of the active regions of the embryo that surround it. First, we find an intermediate characteristic “triangular keyhole” shape in the 3D deformations of the hindgut. We construct a minimal model of the hindgut primordium as an elastic ring deformed by active midgut invagination and germ band extension on an ellipsoidal surface, which robustly captures the symmetry-breaking into this triangular keyhole shape. We then quantify the 3D kinematics of the tissue by a set of contours and find that the hindgut deforms in two stages: An initial translation on the curved embryo surface followed by a rapid breaking of shape symmetry. We extend our model to show that the contour kinematics in both stages are consistent with our passive picture. Our results suggest that the role of in-plane deformations during hindgut morphogenesis is to translate the tissue to a region with anisotropic embryonic curvature and show that uniform boundary conditions are sufficient to generate the observed nonuniform shape change. Our work thus provides a possible explanation for the various characteristic shapes of blastopore-equivalents in different organisms and a framework for the mechanical emergence of global morphologies in complex developmental systems.

## Significance

Deformations of tissues into complex shapes can result from active processes within the tissue or from forces applied at their boundaries. Here, we show that the symmetry-breaking of the shape of the *Drosophila* hindgut primordium that we observe experimentally can be explained by a physical model invoking boundary forces only, with the geometry of the embryo robustly selecting the orientation of this shape. Our mechanism distills the role of the germ band into moving the primordium off the posterior pole and offers an explanation, independent of intratissue forces, for the diversity of observed blastopore-equivalent geometries. More generally, our work introduces the hindgut primordium as a paradigm for understanding intertissue coupling and global morphologies in development.

Morphogenesis can proceed through active mechanisms, which generate tissue deformations by changing cell behaviors within their bounds, or passive mechanisms, which generate deformations via external conditions imposed at their boundaries by neighboring tissues (1). The interplay between active and passive tissues is particularly important during gastrulation, when an embryo has multiple genetically patterned active tissues in addition to passive regions that all deform significantly and almost simultaneously (2).

**Fig. 1.**
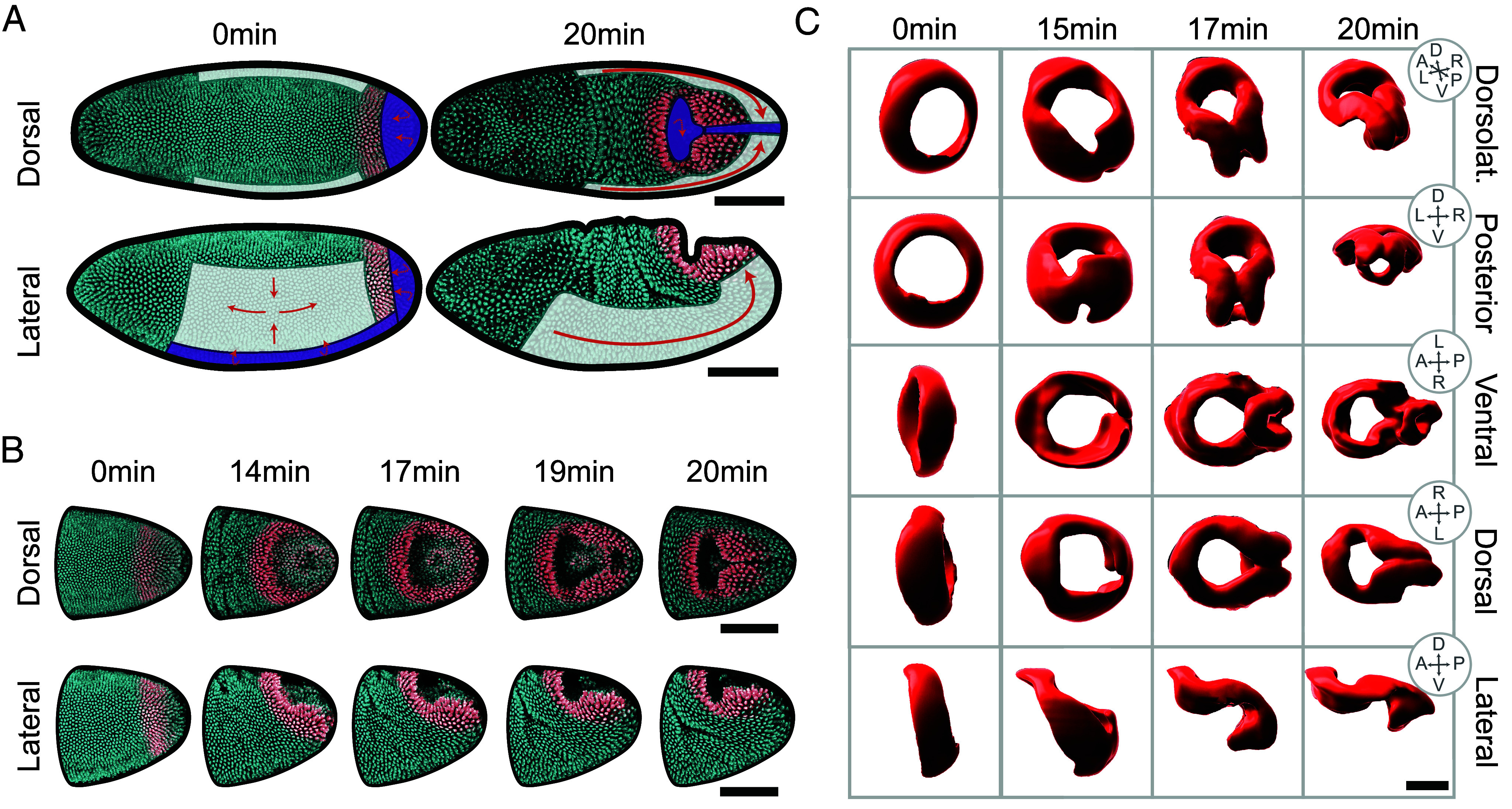
The hindgut primordium is bounded by active tissues and rapidly deforms in 15 min. (*A*) Dorsal and lateral views of the blastoderm at the onset of gastrulation and 21 min later. The cyan signal is a nuclear reporter and the red signal is a nuclear reporter specific to the hindgut (*Materials and Methods*). The germ band, which undergoes in-plane convergent extension, is shaded in white. The ventral furrow, a stripe of cells on the ventral midline, and the posterior midgut, a cap of cells at the posterior pole, undergo out-of-plane invagination and are shaded in purple. Tissue movement is shown using red arrows. (Scale bars: 100 µm.) (*B*) Dorsal (*Top*) and lateral (*Bottom*) views of the deforming hindgut primordium at five timepoints, showing invagination of the posterior midgut as the hindgut deforms into its characteristic triangular shape. (Scale bars: 100 µm.) (*C*) Different views of surface reconstructions of the hindgut primordium from fixed data at timepoints approximated by morphology. (Scale bars: 50 µm.)

Perhaps no developmental system is as well understood as the *Drosophila melanogaster* embryo at the onset of gastrulation, composed of a monolayer of maternally patterned cells between an internal yolk and a vitelline membrane encapsulated by an ellipsoidal rigid chorion. At this stage, several canonical examples of active tissues that are genetically patterned to induce changes in cell shape or activity are undergoing morphogenesis: the posterior midgut (PMG), the ventral furrow (VF), and the germ band (GB) (3, 4, Fig. 1*A*). At the posterior pole, the PMG expresses the transcription factors Huckebein and Tailless (5–7) that signal through the G protein-coupled receptor ligand Fog to activate myosin and induce apical constriction and invagination of the posterior (8–10). Similarly, a stripe of cells in the VF undergoes apical constriction and invaginates to form the mesoderm (11–13). In addition to these out-of-plane deformations, the GB undergoes directed cell–cell rearrangements to converge and extend in-plane, pushing posterior tissue around the posterior pole onto the dorsal side of the embryo (14–18).

While the deformations of these active tissues are striking, they are separated at their boundaries by a domain of cells that deforms no less dramatically, although it lacks obvious expression of genes regulating active deformation (7). This circular domain will ultimately give rise to the hindgut and consists of approximately 450 cells expressing Brachyenteron (*Drosophila* Brachyury). Brachyenteron defines a highly conserved signaling module specifying the posterior fates and gut formation in many organisms (19–22). Homologs include T in mice, No-tail in zebrafish, and XBra in *Xenopus*, and are typically present at the lip of the blastopore-equivalent posterior internalization (23, 24, 25, 26, 27, 28). In *Drosophila*, the domain is ring-shaped and located anterior to the PMG but posterior to the VF and GB (7, Fig. 1*A*). Although Brachyenteron expression is ultimately required for cell-fate-specific differentiation of the hindgut, its elimination has no direct effect on the morphogenetic movements that occur at gastrulation (29). This raises the possibility that early morphogenesis in the hindgut is imposed by forces generated in the surrounding regions.

Embryos provide numerous examples of active deformations in one region exerting forces on neighboring primordia, possibly contributing to their subsequent morphogenesis. Examples of such “boundary-driven” deformations include differential tissue growth driving brain gyrification (30) and vertebrate gut looping (31–33), friction forces driving the first folding event of the zebrafish brain (34, 35) and myotome formation (36), and active contractility at the tissue boundary driving amniote embryogenesis (37, 38). A large body of work has characterized the diverse cellular processes that arise in response to external forces (39), adjacent domains (40, 41), and geometric constraints (42–46), including at the level of individual contributions within a tissue exhibiting both active and passive cellular behaviors (41, 47, 48). However, explanations for global morphological changes of entire passive tissues in the necessary context of their active neighbors and geometric constraints have remained elusive. The *Drosophila* hindgut primordium offers an ideal system to develop a framework for understanding the deformations of such a passive tissue.

In the following experiments, we derive a minimal physical model to investigate whether contributions from adjacent actively deforming tissues and embryonic geometry are sufficient to explain the morphogenesis of the hindgut primordium. We couple our model with 3D imaging of live embryos to quantify the deformations of the hindgut primordium rigorously. We find that as the PMG, VF, and GB impose forces at the boundary of the hindgut primordium, the primordium itself deforms in a combination of in-plane and out-of-plane deformations, breaking the symmetry of its circular shape into a characteristic, intermediate triangular “keyhole” shape (Fig. 1 *B* and *C*). By tracking cells, we reveal a two-stage process and show that the kinematics of both stages are consistent with the passive deformations expected from forces applied at its boundary by the extension of the germ band and the invagination of the midgut that surround it.

## Results

### Description of Hindgut Deformation at Discrete Timepoints.

To visualize the deformations of the hindgut primordium, we used an endogenous fluorescent Brachyenteron protein reporter built on the LlamaTag system (7, 49) to identify the hindgut primordium combined with a standard fluorescently tagged histone nuclear reporter to visualize the entire embryo. Briefly, the LlamaTag system leverages maternally deposited eGFP, which is imported to the nucleus upon the presence of a nanobody fused to the endogenous protein of interest (in our case, Brachyenteron). The deformations of the hindgut primordium were initially visualized using confocal microscopy (*Materials and Methods*).

At the onset of gastrulation, the initially circular tissue deforms significantly in a few minutes (Fig. 1*B*) with no divisions nor cell death, and limited, if any, cell rearrangements (47, 50). The ring initially rotates and translates along the surface of the embryo due to germ band extension (Fig. 1*B*, lateral views at 0 to 14 min) and partially internalizes due to contact with the apically constricting and invaginating posterior midgut (Fig. 1*B*, dorsal views at 0 to 14 min). After this initial phase, the ring rapidly deforms into a characteristic shape (Fig. 1*B*, dorsal views at 17 to 20 min). We will call this shape a “triangular keyhole” because the rotational symmetry of the initial ring breaks to define three regions of higher positive curvature.

To create a more detailed description of these intermediate shapes, wildtype embryos were fixed and stained for Brachyenteron and cell membrane markers Armadillo and Discs large. To visualize the deforming hindgut in 3D at a high isotropic spatial resolution, embryos were imaged using light sheet microscopy (*Materials and Methods* and *SI Appendix*) and staged based on their morphology. Surface reconstructions from the Brachyenteron immunofluorescence signal (Fig. 1*C*) reveal complex intermediate geometries in which the internalized “keyhole” and the triangular shape of the tissue remaining on the surface are more apparent.

### Model of the Symmetry-Breaking of the Hindgut.

**Fig. 2.**
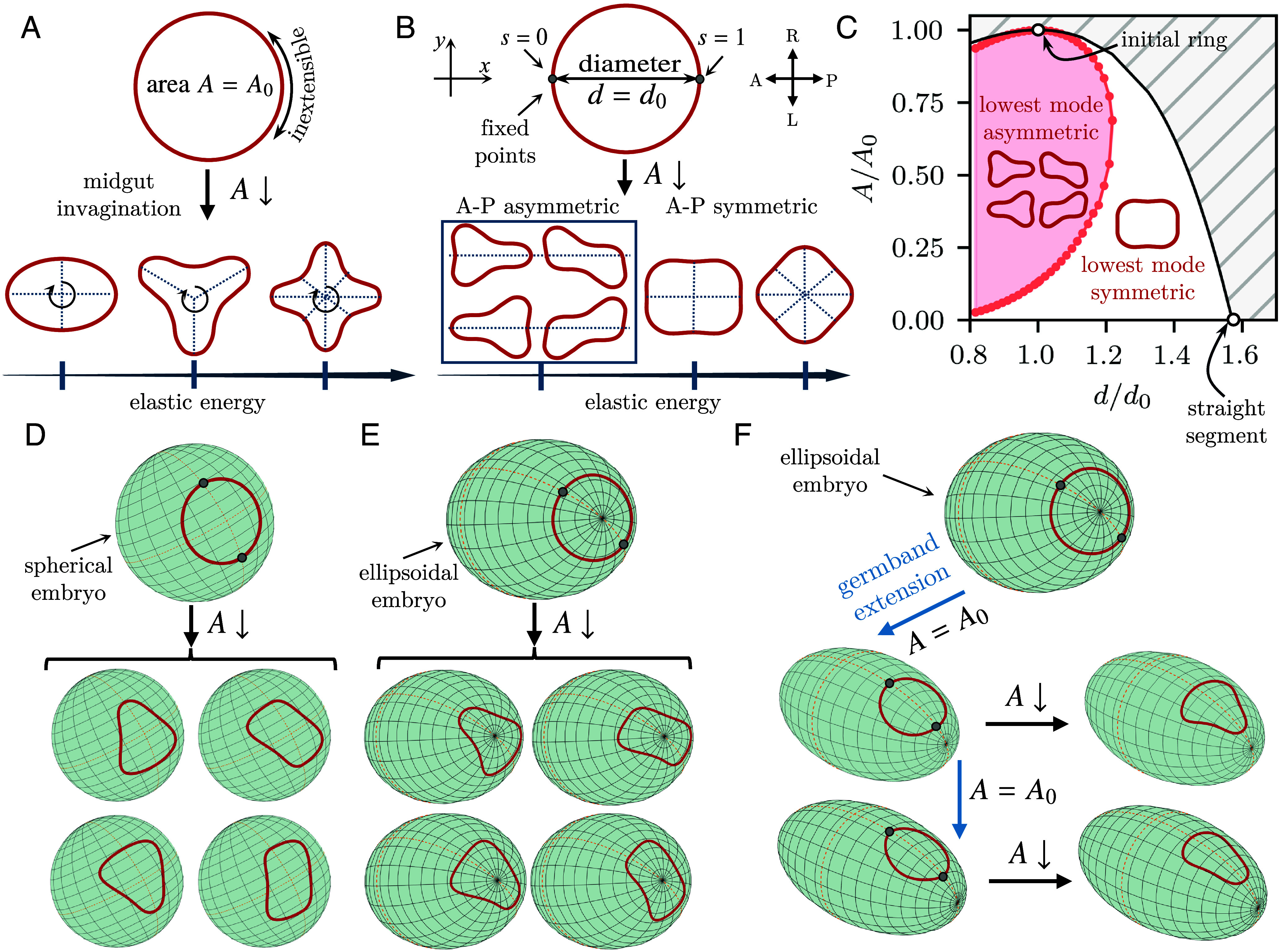
A minimal physical model reproduces the triangular keyhole shape of the primordial hindgut. (*A*) The primordial hindgut is modeled as a planar, inextensible elastic ring enclosing an initial area $A=A_{0}$. Invagination of the midgut reduces the enclosed area to A. The observed shape is the shape of lowest energy and symmetric (51, 52). Additional modes with higher energy also exist and have higher numbers of lobes (51, 52). (*B*) The position of the germ band additionally sets the anteroposterior (AP) diameter d of the ring, i.e., the distance between two diametrically opposite points at arclength positions s=0, s=1. For $d=d_{0}$, the four shapes of equal lowest energy are AP asymmetric, i.e., asymmetric about the y-axis, and include triangular shapes similar to the shape of the primordial hindgut. Additional symmetric and asymmetric shapes are possible as well, but are of higher energies (*SI Appendix*). (*C*) Phase diagram of the bifurcation from panel (*B*) in (d,A) space: The AP asymmetric keyhole shape remains the lowest-energy mode in the shaded region of parameter space as A (midgut invagination) and d (germ band extension) vary. The hatched region is geometrically inaccessible to inextensible deformations. (*D*) An inextensible elastic ring constrained to lie on a sphere breaks symmetry into one of four shapes with equal energies, analogous to the planar shapes in panel (*B*), as the area enclosed by the ring is reduced (midgut invagination) while a diameter is fixed (germ band extension). (*E*) An elastic ring at the posterior pole of an ellipsoid embryo breaks symmetry similarly to the spherical case in panel (*D*). (*F*) Symmetry-breaking of an elastic ring at the posterior pole of an ellipsoid after translation to the dorsal side (germ band extension) and reduction of the area enclosed by the ring (midgut invagination): Among the shapes in panels (*D* and *E*), the gradient in curvature consistently selects the triangular shape with the orientation observed in the *Drosophila* hindgut primordium.

We hypothesized that the shape changes of the hindgut primordium are the passive mechanical consequences of the deformations of the surrounding tissues. We therefore started by deriving a minimal theoretical model of hindgut morphogenesis. In this model, the hindgut primordium is skeletonized to a planar inextensible elastic ring C enclosing an area occupied by the posterior midgut. The ring is initially circular, of area $A=A_{0}$ (Fig. 2*A*). As the midgut invaginates by apical constriction, the effective apical surface area of the tissue decreases, which reduces A and deforms the ring. This deformation minimizes the bending energy of the ring,

[1] $$\begin{array}{c} E=\frac{1}{2}\oint_{C}\kappa {\left(s\right)}^{2}\;\;\text{d}s, \end{array}$$

where s is arclength and κ(s) is curvature, subject to the constraints imposing inextensibility and the area A enclosed by C (*SI Appendix*). This is a well-known mechanical problem (51, 52): The observed shape (of lowest energy) of an elastic ring enclosing a prescribed area is symmetric; higher modes of higher energy have higher numbers of lobes (Fig. 2*A*).

Our minimal model therefore needs one more constraint: The points at which the ring intersects the mid-sagittal cross-section of the embryo cannot move freely, but their position is set at each timepoint by the progress of germ band extension. In the model, this fixes the distance d between two diametrically opposite points on the ring, i.e., its anteroposterior (AP) diameter. The shape of the deformed ring minimizes its bending energy subject to these three constraints. The corresponding Euler–Lagrange equation is

[2a] $$\begin{array}{c} \kappa^{\prime \prime }\left(s\right)+\frac{\kappa {\left(s\right)}^{3}}{2}-\lambda_{0}\kappa \left(s\right)+p=0, \end{array}$$

where dashes denote differentiation with respect to s, and where $\lambda_{0}$ and p are constants to be determined (*SI Appendix*). We complement this with the differential equations

[2b] $$\begin{array}{cccc} & \theta^{\prime }\left(s\right)=\kappa \left(s\right), & x^{\prime }\left(s\right)=\;\cos\theta (s), & y^{\prime }\left(s\right)=\;\sin\theta (s), \end{array}$$

for the tangent angle θ(s) with the AP axis, and the position (x(s),y(s)) of a point on the ring (Fig. 2*B*). The boundary and integral conditions fix the enclosed area to A and the AP diameter to d and impose the symmetry of the half-ring (Fig. 2*B*). They are (*SI Appendix*)

[3a] $$\begin{array}{cc} & \theta \left(0\right)=-\theta \left(1\right)=\frac{\pi }{2},\;x\left(0\right)=y\left(0\right)=y\left(1\right)=0,\;x\left(1\right)=d, \end{array}$$

and

[3b] $$\begin{array}{c} \int_{0}^{1}\left[y(s)\cos\theta (s)-x(s)\sin\theta (s)\right]\;\;\text{d}s=A. \end{array}$$

We solve this boundary-value problem numerically (*SI Appendix*) as A is reduced for $d=d_{0}$, the initial diameter of the ring. The lowest-energy shapes are now asymmetric about the y-axis, i.e., AP asymmetric (Fig. 2*B*). There are four shapes of equal energy, which include “keyhole” shapes reminiscent of the shape of the hindgut primordium. There are also AP symmetric shapes, but they have higher energy (Fig. 2*B*). More generally, d and A both vary as the germ band extends and the midgut invaginates. For inextensible deformations, part of (d,A) space is geometrically excluded. The asymmetric shapes remain the lowest-energy shapes in a large part of the remaining (d,A) space (Fig. 2*C*). This shows that the symmetry-breaking bifurcation that can lead to triangular shapes is robust to variations of $d\neq d_{0}$.

### Selection of Hindgut Shape by Embryonic Curvature.

The four-fold degeneracy of the shapes of minimal energy in Fig. 2*B* raises the question: How does the embryo consistently select one of these orientations? To answer this, we extended our model of a planar ring to a spherical or ellipsoidal surface approximating the embryonic geometry. However, even for these simple curved surfaces, the equation analogous to Eq. **2** becomes too complex to write down. Instead, we directly minimized the bending energy in Eq. **1**, subject to the same constraints, for shapes approximated by a few Fourier terms (*SI Appendix*). An elastic ring on a sphere (Fig. 2*D*) or at the posterior pole of an ellipsoid (Fig. 2*E*) still breaks symmetry as A is reduced, but the shape degeneracy persists by symmetry. If, however, the ring translates off the posterior pole and onto one side of the ellipsoid (similarly to the translation of the hindgut primordium onto the dorsal side of the embryo due to germ band extension), then the curvature gradients eliminate the degeneracy and the ring selects a triangular shape in the same orientation as the shape of the hindgut primordium (Fig. 2*F*).

Our minimal model thus shows that uniform contraction, representing midgut invagination, is sufficient to explain the symmetry-breaking of the hindgut primordium, with the observed shape selected by the curvature of the embryonic surface. In particular, neither active deformations of the hindgut primordium, nor inhomogeneous forces from the extending germ band that surrounds it, nor heterogeneities in its passive mechanical properties are necessary to explain the triangular shape qualitatively.

### Real-Time Kinematics Inferred from Live Imaging.

**Fig. 3.**
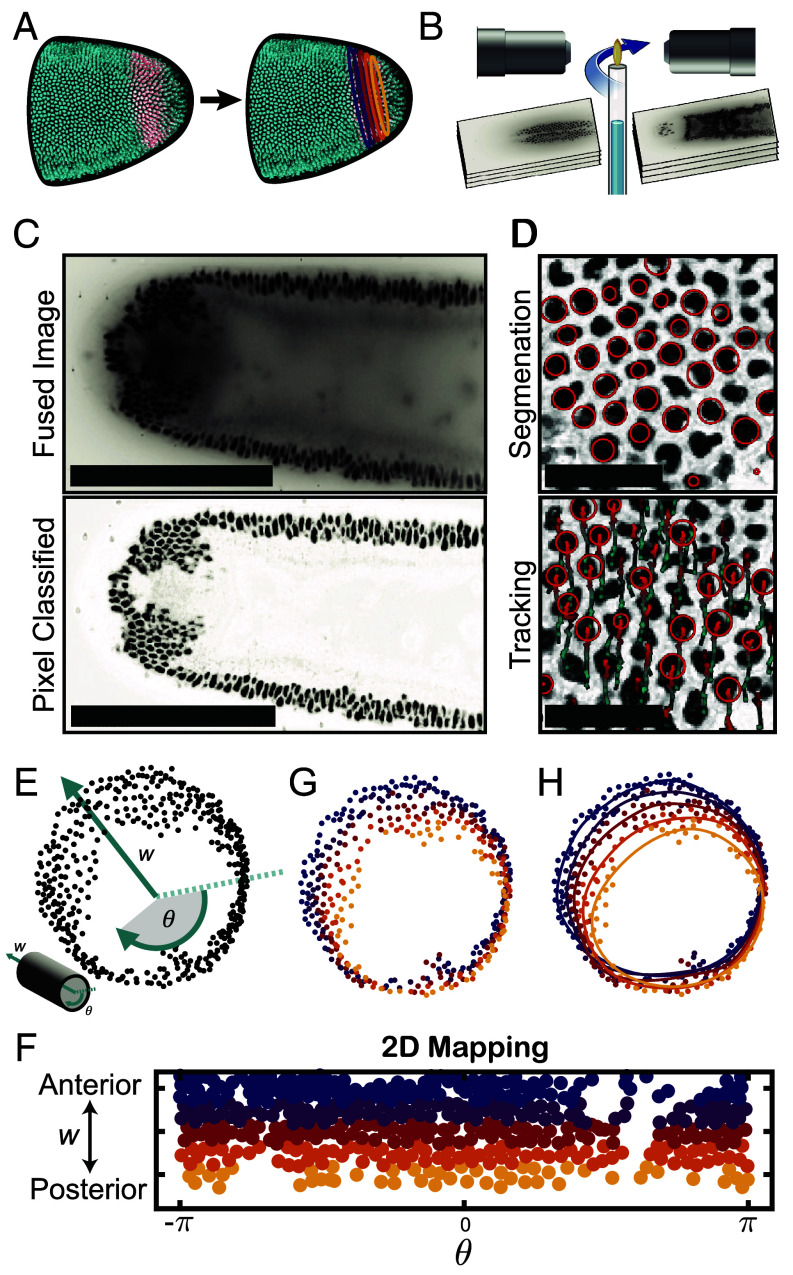
Data analysis pipeline. (*A*) The analysis constructs a set of closed space curves (“contours”) that are initialized by positions of nuclei within the hindgut primordium and deform with it in time. (*B*) Light sheet microscopy enables simultaneous imaging of both sides of embryos with fluorescent reporters for nuclei and hindgut. (*C*) After image fusion and deconvolution (*Materials and Methods* and *SI Appendix*), images are processed using a pixel classifier (ilastik, 53) to improve nuclear detection. (Scale bars: 200 µm.) (*D*) Nuclei within the hindgut primordium are segmented into spots (*Top*); these spots are tracked semiautomatically using Mastodon (54) (*Bottom*) to generate a full track for each nucleus in the hindgut primordium. (Scale bars: 20 µm.) (*E*) Initial positions of nuclei at the blastoderm stage are mapped into cylindrical axial and angular coordinates w,θ (*Inset*). (*F*) Nuclei are binned into contours by their anteroposterior position w in this 2D mapping. (*G*) Initial nuclear positions from panel (*E*) colored by the contour to which they are assigned from the binning in panel (*F*). (*H*) Contours are fitted using a sequence of splines that update at each timepoint as the nuclei move. Here, the initial contours are overlaid on from panel (*G*).

To understand the kinematics of the hindgut primordium, we generated a series of closed space curves that we term “contours.” Contours track the movement of nuclei within the hindgut primordium during the first 20 min of gastrulation (Fig. 3*A*) and visualize the deformations of the hindgut primordium as threads on the surface of a fluid visualize its flow. First, we used light sheet microscopy to image (*Materials and Methods*) the deforming hindgut (Fig. 3*B*). We cooled the embryos to slow development, increasing the effective temporal resolution, and generated a 4D dataset with isotropic spatial resolution in one or two channels at a time resolution of 6 to 10 s. After fusing and deconvolving images, we classified pixels using a standard tool (53) to remove fluorescence from the yolk and beads used to register the images (Fig. 3*C* and *SI Appendix*). Pixel-classified images were segmented using a difference-of-Gaussians detector that approximates nuclei as 3D spheres (55, 56, Fig. 3*D*). We tracked nuclei semiautomatically in Mastodon (54), a tool built on the TrackMate (55, 56) plugin for Fiji (57). Each nuclear track was manually verified or corrected, resulting in approximately 500 tracks over approximately 100 timepoints (Fig. 3*D*). We initialized contours by mapping the initial nuclear positions at the blastoderm stage into cylindrical coordinates (Fig. 3*E*). Nuclei were binned into five groups based on their cylindrical axial coordinate w, corresponding to their embryonic anteroposterior positions (Fig. 3*F*). Doing so divides the ring of the hindgut primordium into five slices (Fig. 3*G*). Contours were fitted to each of these slices using a series of splines (Fig. 3*H*). Contours were continually refitted using bins propagated from the initial assignments to capture the updated nuclear positions at subsequent timepoints (*Materials and Methods* and *SI Appendix*), revealing the kinematics of the developing hindgut (Movie S1).

### Two Stages of Hindgut Morphogenesis.

**Fig. 4.**
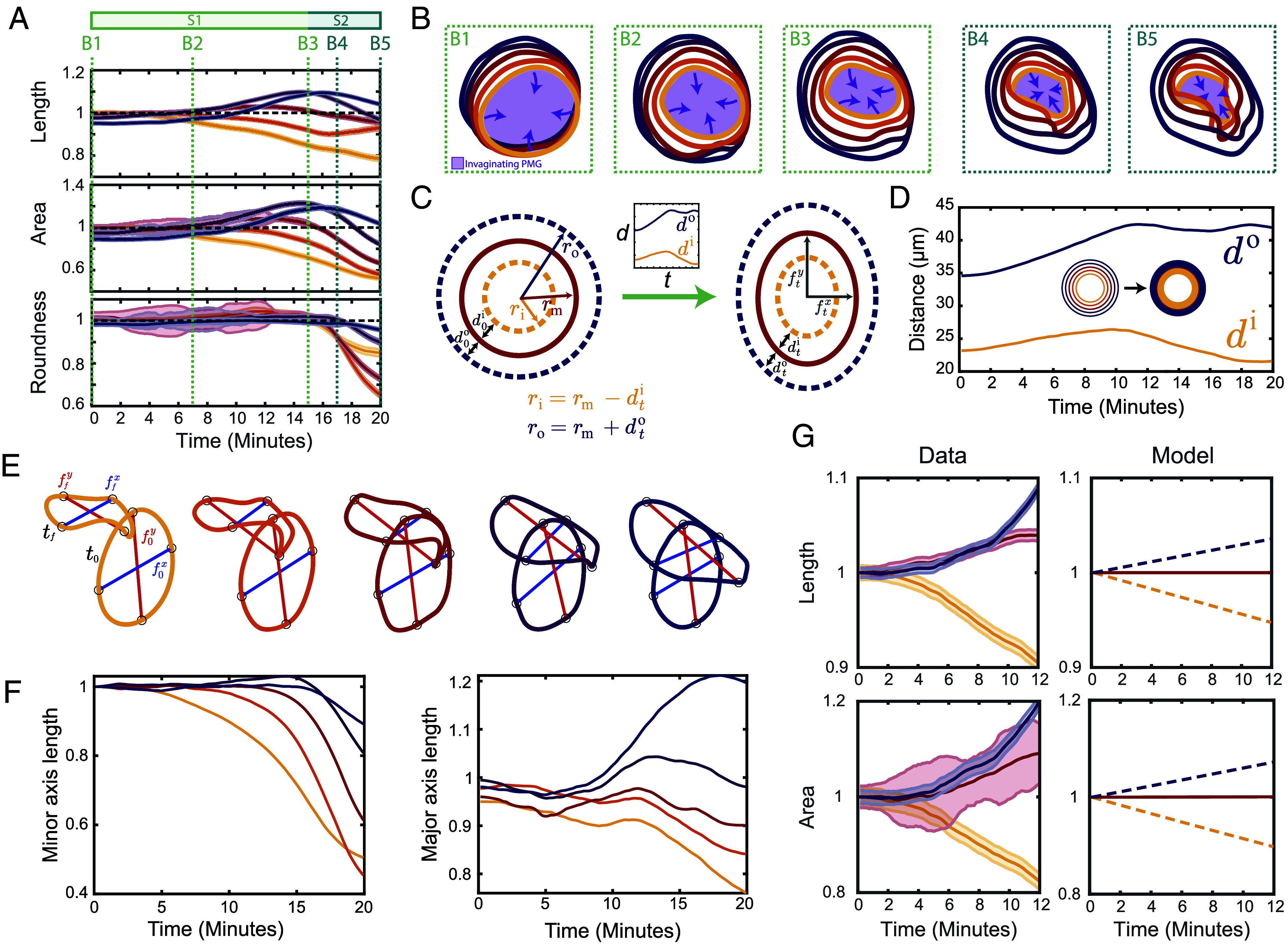
The hindgut primordium deforms in two stages. (*A*) Shape metrics (contour length, enclosed area, and roundness) plotted against time for a representative embryo, colored by contour (innermost, yellow to outermost, blue). There are two stages: During stage S1 (green), all contours maintain their initial roundness and the lengths and areas of the inner and outer contours decrease and increase, respectively. From t=15min onward (stage S2, turquoise), the areas enclosed by all contours decrease and the roundness of all contours but the outermost one decreases sharply. Dashed lines, colored by stage, indicate timepoints *B1*–*B5* used in panel (*B*). Error bars are determined from the SD of a simulated error distribution (*SI Appendix*). (*B*) Contour shapes at the timepoints *B1*–*B5* highlighted in panel (*A*). The violet shading indicates the invaginating posterior midgut (PMG). (*C*) “Coupled-ring” model of the deformation of circular contours into ellipses (*SI Appendix*). At time t, the middle contour has semiminor axis $f_{t}^{x}$ and semimajor axis $f_{t}^{y}$, and the initial distances $d_{0}^{\text{i}},d_{0}^{\text{o}}$ from the middle to the inner and outer contours have changed to $d_{t}^{\text{i}},d_{t}^{\text{o}}$, respectively (*Inset*). (*D*) Plot of the measured mean distances $d^{\text{i}},d^{\text{o}}$ from the middle to the innermost and outermost contours (*SI Appendix*) against time. *Inset*: The contours define inner and outer rings used for calculating $d^{\text{i}},d^{\text{o}}$. (*E*) Definition (*SI Appendix*) of the minor (blue) and major (red) axis lengths $f_{t}^{x},f_{t}^{y}$, shown for each contour at the initial and final timepoints $t_{0},t_{f}$. (*F*) Plots of the minor and major axis lengths or each contour, normalized by their initial lengths, against time. (*G*) The “coupled-ring” model (*Right*, *SI Appendix*) sketched in panel (*C*) explains the qualitative kinematic behavior of the inner and outer contours during stage S1 (*Left*): If the length (*Top*) or area (*Bottom*) of the middle contour is constant (solid line), the model predicts (dashed lines) that the lengths or areas of the inner and outer contours decrease and increase, respectively, consistently with the data (*Left*).

To quantify the contour kinematics, we computed shape metrics at each timepoint and plot the normalized length, area, and roundness of each contour in Fig. 4*A* for a representative embryo. The length of the middle contour changes minimally over the first twenty minutes of gastrulation, which is consistent with the approximation of an inextensible midline and use of an elastic description (as opposed to viscous description permitting cell rearrangements) in our physical model of the symmetry-breaking. Moreover, this quantification reveals that the deformation has two stages (Fig. 4*A*).

During the first stage, the area and length of the outer and inner contours increase and decrease monotonically, respectively, while the area enclosed by the middle contour displays little to no change. The roundness of each contour remains close to unity, indicating uniform dilation and compression of the contours. Qualitatively, the shapes of all contours remain elliptical and begin to rotate and translate along the surface of the embryo as gastrulation begins (Fig. 4*B1* and *B3*). Toward the end of the first stage, apical constriction of the posterior midgut causes the areas enclosed by the contours to begin to decrease, starting with the innermost contour adjacent to the posterior midgut.

The second stage involves a sharp decrease of the roundness of all contours, with the outer contours remaining rounder than the middle and inner contours (Fig. 4*A*). As the contours move up and around the posterior pole (Fig. 4*B4* and *B5*), the midgut fully involutes and inverts, causing the areas enclosed by each contour to decrease (Fig. 4*A*). The contour lengths display more complex behavior, likely due to the out-of-plane deformations of the deforming hindgut. Interestingly, the inner contours, initially closer to the posterior, start to decrease in length, area, and roundness slightly before the outer contours. We computed the same metrics in terms of the position of the ring along the embryonic surface (*SI Appendix*, Fig. S5*A*), observing that all three shape metrics start to decrease when the ventral-most point of the contour passes the posterior pole (*SI Appendix*, Fig. S5*B*). This suggests that the delay results from different contours occupying similar regions of the embryo at slightly different times.

### Minimal Geometric Model of the Observed Contour Kinematics.

To explain the contrasting changes in the lengths and areas of the inner and outer contours during the first stage qualitatively, we introduced a minimal “coupled–ring” model describing an inner, middle, and outer contour (Fig. 4*C* and *SI Appendix*). We hypothesized that the changes of the inner and outer contours are a consequence of the smaller deformations of the middle contour (which becomes slightly elliptical) and of the changes of its distance to the inner and outer contours. We therefore quantified (*SI Appendix*) the mean distance between contours (Fig. 4*D*) and their major (anteroposterior) and minor (*Left*/*Right*) axis lengths (Fig. 4 *E* and *F*). To explain the relative behaviors of the inner and outer contours, we first modeled the length of the middle contour to be constant because of its lesser length change during stage S1. This predicts that the lengths of the inner and outer contours decrease and increase, respectively (Fig. 4*G*). Similarly, assuming that the area of the middle contour is constant, the model shows a decrease and increase of the inner and outer contour areas, respectively (Fig. 4*G*). The “coupled-ring” model thus captures the observed qualitative kinematics of the innermost and outermost contours. Quantitative differences do arise, however, likely because of out-of-plane deformations.

Interestingly, the major axis (i.e., the anteroposterior diameter) of the outermost contour has lengthened significantly by the end of the process (Fig. 4*F*), while the major axes of the other contours remain constant or shorten. At the same time, the shape of the outermost contour remains roundest (Fig. 4*A*). Only the middle and inner contours adopt the triangular shape that we have predicted in our minimal planar model. This is consistent with our model because shapes do not break symmetry if their anteroposterior diameter increases too much, as is the case for the blue outermost contour (Fig. 2*C*).

### Model Verification Using Genetic Perturbations.

**Fig. 5.**
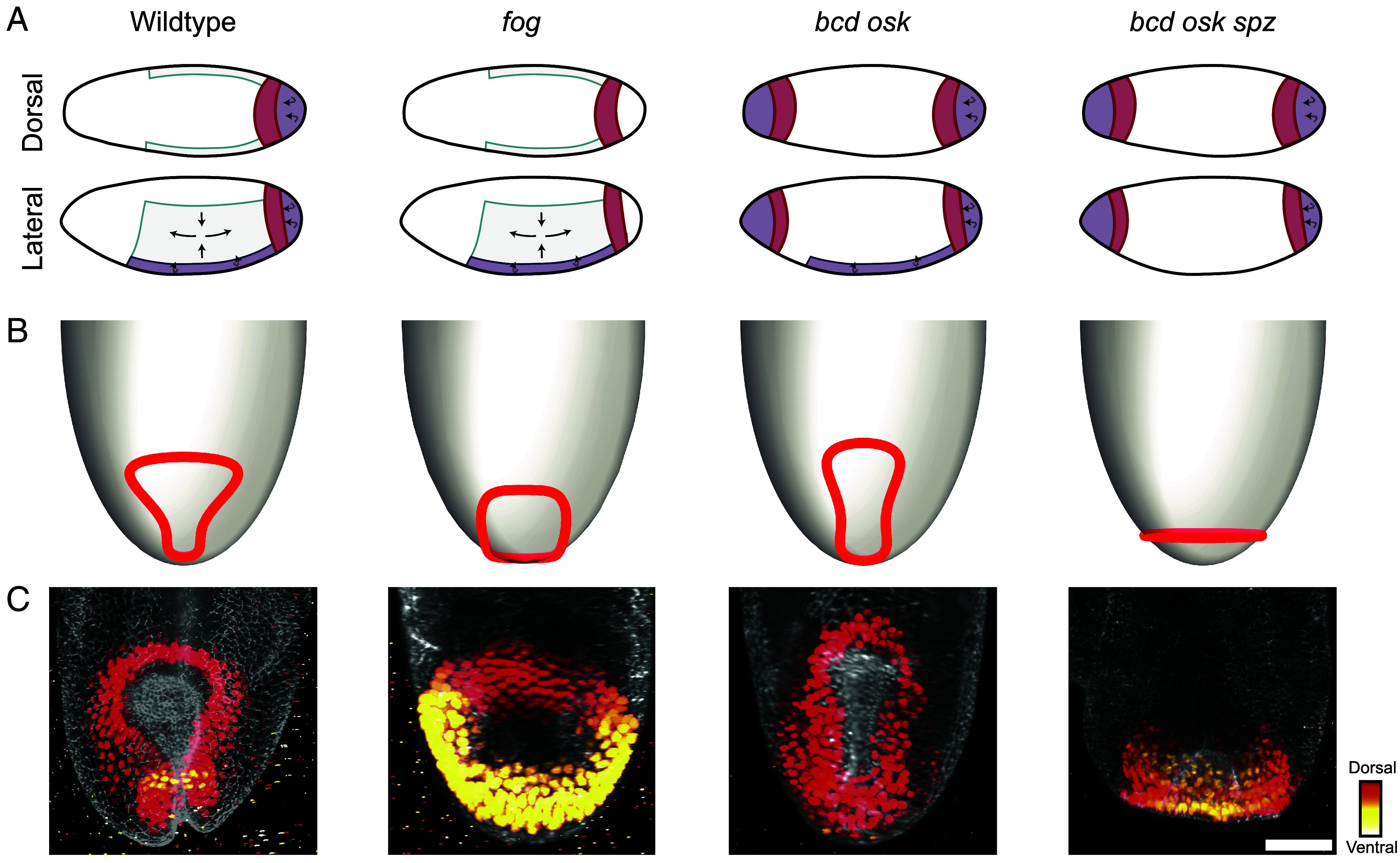
Comparison of model predictions with genetic perturbations. (*A*) Schematics showing the simplified effects of the different perturbation compared to the wildtype (WT). The germ band is shaded in cyan, the ventral furrow and the midgut(s) in purple, and the hindgut(s) in red. Arrows indicate directions of tissue movements, similarly to Fig. 1*A*. In *fog* mutants, apical constriction within the posterior midgut is absent. In *bcd osk* mutants, the anterior terminal domain shows a duplication of posterior terminal fate, the decrease in enclosed area appears unchanged from WT, there are no active cell intercalations within the germ band, and dorsal contraction is preserved. In *bcd osk spz* mutants, in addition to the absence of germ band extension and a duplicated posterior, the embryo trunk adopts a uniform dorsalized fate, leading to uniform dorsal contraction around the ring. (*B*) Model predictions of the shape of the hindgut in WT (Fig. 2*F*) and in these mutants from numerical simulations. In the *fog* mutant, the ring remains closer to the posterior pole than in WT and the enclosed area does not decrease. In the *bcd osk* mutant, the enclosed area decreases similarly to WT and translation onto the dorsal surface is preserved due to dorsal contraction, which also causes the ring to stretch slightly more along the anteroposterior axis than in WT. In the *bcd osk spz* mutant, the area enclosed by the ring decreases but the ring remains at the posterior pole due to uniform dorsal contraction around it. (*C*) Maximum intensity projections of dorsal views of fixed WT and mutant embryos, confirming the model predictions. Brachyenteron signal is color-coded according to depth along the dorsoventral direction. (Scale bar: 50 µm.)

Our minimal model proposes that the symmetry breaking of the shape of the hindgut results from two necessary mechanisms: a decrease in enclosed area and a translation of the ring to a region of anisotropic curvature. In order to test these hypotheses, we turned to established and well-characterized genetic perturbations (Fig. 5*A*).

In embryos mutant for *folded gastrulation* (*fog*), posterior midgut cells do not undergo apical constriction and germ band extension is reduced (Fig. 5*A*). In the model, this corresponds to the ring remaining close to the posterior pole, while its enclosed area does not reduce. The resulting shape predicted by the model, from numerical minimization of the bending energy, is an approximately circular ring (Fig. 5*B*). Maximum intensity projections of light sheet microscopy datasets of fixed *fog* embryos confirm this prediction (Fig. 5*C*).

In *bicoid oskar* (*bcd osk*) mutants, the anterior terminal region of the embryo duplicates the posterior terminal fate, leading to two hindgut primordia (Fig. 5*A*). The posterior midgut undergoes normal apical constriction, but active cell intercalations within the germ band are absent, abolishing germ band extension. Dorsal contraction, a morphogenetic force orthogonal to germ band extension, is sufficient to pull the ring onto the dorsal side even in the absence of active germband extension (14). The model therefore predicts the ring to break symmetry, and the observed hindgut shape in *bcd osk* mutants (Fig. 5*C*) is consistent with the shape predicted by the model even for an increased value of d from dorsal contraction (Fig. 5*B*).

Finally, in embryos mutant for *bicoid oskar spätzle* (*bcd osk spz*), the trunk is uniformly dorsalized, resulting in uniform dorsal contraction around the ring (Fig. 5*A*). In these embryos, the midgut continues to undergo apical constriction but there is no movement of the ring from the posterior pole. The model now predicts that this shape will buckle slightly without a persistent axis of bilateral symmetry (Fig. 5*B*). The mutant morphology shows a slightly contracted circular hindgut primordium, but no keyhole, consistent with predictions (Fig. 5*C*).

In particular, the hindgut morphologies observed in the *fog* and *bcd osk spz* mutants indicate that both a decrease in enclosed area and translation from the posterior pole are required for symmetry breaking, in agreement with our model. Moreover, the *bcd osk* mutant suggests that any biological mechanism that translates the ring off the posterior pole can satisfy the condition of our model that the ring be translated to a region of anisotropic curvature.

## Discussion

Any developmental system composed of both actively deforming and passive tissues (30, 31, 34, 35, 37, 38) inevitably features deformations in boundary regions bridging actively deforming neighbors. Such “boundary-driven morphogenesis” has proven difficult to understand, even at the level of kinematics, due to complex combinations of in-plane and out-of-plane deformations. This difficulty is compounded by the facts that boundary-driven and active morphogenesis can combine within the same tissue and that different combinations of passive and active cell behaviors can generate similar tissue deformations (40, 41, 47). We have shown that our understanding of the morphogenesis of the *Drosophila* hindgut primordium, both in wildtype as well as in different genetic mutants, is consistent with a minimal model in which its complex deformations result solely from the forces exerted by its actively deforming neighboring tissues and the ellipsoidal geometry of the eggshell. Its dramatic change in shape, well-characterized neighboring tissues, and compatibility with well-established techniques for *Drosophila* cell biology make the hindgut an ideal model for boundary-driven morphogenesis.

Previous work has described specific cellular processes in embryonic primordia ranging from the internalization of cells in the mesoderm (48, 58–60) to biased cell rearrangements in the germ band (41, 43, 47), proposing critical insights into how deformations may occur. Ultimately, fully understanding morphogenesis requires a more global approach that can integrate these individual findings. Here, we have taken such an approach that has allowed us to examine the full deformation of the hindgut primordium in its biological context. Our mechanism depends only on a uniform reduction of apical area by invagination of the posterior midgut and a uniform boundary condition from the germ band that translates the ring off the posterior pole. Movement of the ring to a region where the eggshell imposes anisotropic embryonic curvatures resolves the degeneracy of this symmetry-breaking and selects a triangular shape with proper orientation. Our minimal model absorbs these complex in-plane and out-of-plane deformations into simplified yet biologically relevant and measurable parameters, including the area enclosed by the tissue and its anteroposterior diameter. This paradigm will also be able to resolve which physical effects are likely to drive the observed global morphological changes in other developmental processes with complex boundary conditions.

Although we have distilled the complex 3D shape of the hindgut that we observed in Fig. 1*C* into a triangular shape on the surface of the embryo, future work will need to understand the out-of-plane deformations of the internalized “keyhole” shape where the propagating ventral furrow meets the involuted midgut (Fig. 1*C*). In addition, we observed some in-plane stretching of the tissue between the contours in the anteroposterior direction, as evidenced by the changing intercontour distances (Fig. 4*D*). Further work will need to resolve the mechanical basis for this deformation within the hindgut. Continuum mechanical approaches (61) will enable elucidating the contributions of in-plane and out-of-plane boundary conditions from the neighboring active tissues to these and other characteristics of the hindgut shape. This will be aided by the rapid advances in techniques for measuring passive tissue properties (62–67), perturbing cytoskeletal elements (68, 69), and machine-learning-assisted computer vision (70–73), all of which will ultimately be used to populate a descriptive atlas of morphogenesis (74). This approaching wave of data will couple to our framework to resolve mechanisms for global morphologies in development.

More generally, by demonstrating the possible role of embryonic curvature in selecting the orientation of the triangular shape of the hindgut primordium, our work also offers an explanation for the effect of embryonic geometric constraints on the morphogenesis of other tissues. In many organisms, Brachyury is expressed at the lip of the blastopore or a similar invaginating structure (19, 22, 24, 75) that deforms into various shapes depending on the organism. In some of these organisms, the blastopore lip appears as a constricting ring on a spherical embryo that fluidizes through cell rearrangements or oriented divisions, which can relieve stresses imposed at the boundaries through internal viscous dissipation (76–79). In some insects with more elongated embryos than those of *Drosophila*, such as the medfly, germ band extension and posterior invagination differ, yet the lip of the posterior invagination also looks triangular as it moves off the posterior pole (80). In the beetle *Tribolium castaneum*, the serosa undergoes epiboly through a mechanism separate from germ band extension and forms an intermediate triangular window on the ventral side of the ellipsoidal embryo (81, 82). Using our framework to understand the mechanisms that drive the emergence of blastopore shapes will provide further insights into the evolution of the blastopore-to-primitive streak transition (83, 84).

More physically, our triangular shape bifurcation expands the large body of work on constrained elastic lines in the plane and on curved surfaces (51, 52, 85–92) and related problems (93–96). In this context, the shape-selection mechanism that we propose stresses the importance of anisotropic curvature for such bifurcations. The hindgut primordium and the ellipsoidal *Drosophila* embryo more generally therefore provide a paradigm for mechanical bifurcations within curved surfaces. Indeed, very recent work has shown that even the minimal instability that is Euler buckling changes fundamentally within general curved surfaces (97), but, compared to the well-understood instabilities of curved surfaces (30, 98–105), these instabilities within curved surfaces remain mysterious.

## Materials and Methods

### Imaging and Tracking.

In order to visualize nuclei and identify hindgut progenitors, we generated a line containing the histone tag Histone H2B-RFP with the maternal ubiquitously expressed GFP under the *bicoid* promoter. Females from this stock were crossed with males containing the previously generated Brachyenteron LlamaTag (7, 49). To generate the movie stills showing lateral and dorsal views of the deforming hindgut, embryos were manually dechorionated on double-sided tape before being immersed in halocarbon oil on custom filter slides and imaged using a Leica SP5 scanning confocal microscope. For tracking, embryos were manually dechorionated on double-sided tape and mounted in capillary tubes containing a solution of 1% agarose with 1:200 diluted TetraSpeck 0.2 µm microspheres (ThermoFisher #T7280). Imaging was performed on a Bruker/Luxendo MuVi-SPIM light-sheet microscope at 33.3× magnification using two cameras mounted opposite each other and a rotating stage (Fig. 3*B*). Syncytial embryos were cooled to $18^{{}^{\circ}}$C and full stacks were taken in the sagittal and frontal planes and in two channels (nuclei and Byn reporter) every 60 s to monitor the progression of development and designate cell identities. At the onset of gastrulation, defined as the onset of ventral furrow formation, imaging was switched to a single image stack acquired through the frontal plane (through the dorsoventral axis) in the histone reporter channel every 7.75 s at slice thicknesses of 1 µm to maximize temporal resolution. After 12 to 18 min, the imaging mode was switched back to the initial 2-channel, 2-angle mode to monitor further development. Embryos with visibly aberrant development or arrest were discarded from the dataset. Nuclei were tracked using Mastodon (54 and *SI Appendix*).

### Construction of Contours from Data.

Raw tracks were smoothed by using an exponential moving average filter on each spatial dimension with a window size of 10 timepoints, or 80 to 110 s. Only nuclei that could be tracked through each timepoint were used. Approximately 5 to 10% of nuclei, typically contained within the ventral midline in the ventral furrow, could not be tracked reliably throughout the full movie. To initialize contours, positions of nuclei at the first timepoint were mapped into cylindrical coordinates (Fig. 3*E*). Positions were first normalized and then projected into eigenspace using a correlation matrix. Coordinates in eigenspace were converted to cylindrical coordinates, of which only the polar angle and the axial coordinate were used for mapping. Nuclei were binned into 5 bins based on their axial coordinates, corresponding to bands 2 to 3 nuclei wide to be used to fit contours. Each bin defined an initial contour identity, and these were propagated forward in time as nuclear positions changed.

To generate a contour at a given timepoint, points within the corresponding bin were sorted based on their initial azimuthal angle and their updated spatial coordinates were repeated three times to reduce edge effects. A cubic smoothing spline was applied to each dimension using the csaps function in MATLAB (The MathWorks, Inc.) with a smoothing parameter of 0.01. To extract a single closed contour, we iterated simultaneously in the forward and backward directions from the midpoint of the repeated array that contains the knots of these splines until these knots fell within a fixed tolerance of each other (which indicates completion of a full loop). A closed space curve was then obtained by joining the two knots and discarding knots outside of the interval containing the midpoint. All contours were resampled to generate space curves of 500 kn with constant arclength spacing.

For visualization, points in 3D were projected onto the same 2D dorsolateral viewing plane for all figures except Fig. 4*E*, which uses an alternative viewing plane to better differentiate major and minor axes.

### Calculation of Shape Metrics.

The length L of a contour was computed as the sum of the arc lengths of each spline within that contour. Its area A was calculated by identifying its dorsalmost and ventral-most points to creating a line of bilateral symmetry. From this, the area was obtained as the (Riemann) sum of the lengths of line segments between corresponding points on either side of this midline multiplied by the distance between them. The roundness was defined to be $R=A/L^{2}$. In Fig. 4, each of these metrics was normalized by its value when the ventral-most point of the respective contour was located at a reference position. For this purpose, we first approximated the surface of the embryo by an ellipsoid with aspect ratio 2.5:1:1, based on the aspect ratio 185 µm:92.5 µm:92.5 µm of the representative embryo used for Fig. 4 measured using Fiji (57). This reference position was then chosen to be the initial position of ventral-most point of the innermost (yellow) contour. To obtain metrics in terms of the positions of the contours (*SI Appendix*, Fig. S5), we reparameterized contours similarly by the positions of their ventral-most points along the arclength of a sagittal cross-section of this ellipsoid, s(θ)=E(θ,ε), where E(θ,ε) is the incomplete elliptic integral of the second kind, ε=0.92 is the eccentricity of this elliptical cross-section, and θ is the polar angle measured from the anteroposterior axis.

### Additional Experimental and Image Analysis Methods.

Further details of the experimental and image analysis methods are given in *SI Appendix*.

### Physical Models.

Details of the derivations of the physical models are given in *SI Appendix*.

## Supplementary Material

Appendix 01 (PDF)

Movie S1. Contour dynamics. Contours are shown updating in time with smoothed nuclear positions visible as points. The nuclei are colored by the contour to which they belong. The movie shows the first 20 minutes of gastrulation.

## Data Availability

Code has been deposited in Github https://github.com/dralber/HindgutContours (106) and microscopy data have been deposited in the BioImage Archive https://doi.org/10.6019/S-BIAD1689 (107).
